# An Improved WMS-*2f/1f* Spectral Fitting Method Using Orthogonal Test in Initial Parameters Selection

**DOI:** 10.3390/s22197430

**Published:** 2022-09-30

**Authors:** Liezhao Luo, Ting Li, Jiangge Deng, Runzhou Zhao, Jinkui Wang

**Affiliations:** 1 School of Energy and Power Engineering, Beihang University, Beijing 100191, China; 2 National Key Laboratory of Science and Technology Aero-Engine Aero-Thermodynamics, Beihang University, Beijing 102206, China

**Keywords:** TDLAS, wavelength modulation spectroscopy, orthogonal test, spectral fitting

## Abstract

This paper proposes an improved wavelength modulation spectroscopy with the 2nd harmonics normalized by the 1st harmonics (WMS-*2f/1f*) spectral fitting method using the orthogonal test in selection of the initial parameters. The method is implemented and validated experimentally in measurement of the temperature of diluted H_2_O in air (1 atm, 291K, 0.7%) by the WMS-*2f/1f* technique. The transition center wavelength targets near 1344 nm. Results demonstrate that the sum-square-error (SSE) between the calculated and measured WMS-*2f/1f* spectral profiles decreases significantly within given updating times when the optimized initial parameters are used. Compared to the conventional method, the optimized initial parameters can make the fitting routine converge more efficiently. The temperature of the vapor inferred from the proposed spectral fitting method are in good agreement with the true values.

## 1. Introduction

The tunable diode laser absorption spectroscopy (TDLAS) has drawn considerable attention in the last few decades. Due its high sensitivity, accuracy and non-intrusive characteristics, it has been applied in various fields [1,2,3,4,5,6,7,8,9,10,11,12,13]. The TDLAS techniques can be categorized into direct absorption spectroscopy (DAS) and wavelength modulation spectroscopy (WMS). The former is relatively straight forward but susceptible to the harsh environment. The latter is complicated in the theory and data process but it can achieve improved noise-rejection ability and sensitivity [14]. In WMS, the laser is scanned by a low-frequency waveform *f*_s_ and a high-frequency sinusoidal waveform *f*_m_ simultaneously. Therefore, the absorption information is conveyed to the high frequency harmonics. In order to decouple the absorption information from harmonics, several strategies have been developed [15,16,17,18,19,20,21,22]. Among them, the scanned-WMS-*nf/1f* fitting method proposed by Sun et al. [19] and developed by Goldenstein et al. [14] is effective for all kinds of situations and avoids the complex analytic expression [16,18], which makes it prevail in the WMS data process. In this scheme, the absorption information is retrieved from harmonics by fitting the simulated WMS-*nf/1f* spectral profile to the measured one proceeded with the same digital lock-in amplifier. Specifically, the spectral parameters, such as the integrated absorbance area, *A*, the transition center frequency, $v_{0}$ and the lineshape width, Δν, are used as the free values in the fitting routine. If the sum-square-errors (SSE) between the simulated WMS-*nf/1f* profile and the measured one reach to the predetermined value, the outputs of the routine are employed to infer the gas properties. Nevertheless, it may take a long time to obtain the satisfied results when the initial parameters are chosen inappropriately, for the efficiency of the fitting routine is heavily dependent on the values of initial parameters. However, the investigation of the initial parameter optimization in the WMS-*nf/1f* fitting is rather limited.

The orthogonal test method (OTM) is based on the orthogonal table to analyze the experiment results scientifically [23]. It has been applied widely in optimizing the experimental scheme, which can reduce testing numbers, shorten test time and minimize cost [24,25,26,27,28]. In this study, the WMS-*nf/1f* fitting performance is dependent on the initial parameters, namely the integrated absorbance area, *A*, the transition center frequency, and $v_{0}$, the lineshape width, Δν, which is similar to the multifactor and multilevel problem in the orthogonal test method. Therefore, it is possible to optimize the initial parameters with the orthogonal test method to enhance the fitting efficiency.

In this paper, the optimization of initial parameters was performed based on the orthogonal test method. The optimized initial parameters were used to match the measured WMS-*2f/1f* spectral to infer the gas properties, which decreased the SSE between the calculated WMS-*2f/1f* spectral profile and the measured one within the given updating times and shortened the fitting period, accordingly, enhancing the fitting routine efficiency.

## 2. Theory

### 2.1. Wavelength Modulation Spectroscopy

The basic principle of WMS has been described in detail in large quantities of other literature [29,30,31,32]. Here, a brief review is presented. All the absorption spectroscopy techniques are governed by the Beer-Lambert law that relates the transmitted and the incident light at frequency, *v*, as described in Equation (1)

(1) $$I_{t}{}_{,v}=I_{0,v}\exp(-\alpha_{v})=I_{0,v}\exp(-S(T)\phi_{v}PX_{abs}L)$$

where $I_{t,v}$ and $I_{0,v}$ are transmitted and incident light intensity at frequency, v, respectively. $\alpha_{v}$ is the spectral absorption at frequency v. S(T) (cm^−2^atm^−1^) is the linestrength, which is dependent on temperature only. *P* (atm) is the total pressure of the absorbing species and *X_abs_* is the mole fraction of the absorbing species. $\phi_{v}$ represents the line shape function and *L* (cm) is the optical length.

WMS is an advanced TDLAS technique based on direct absorption spectroscopy (DAS), which has a higher signal noise ratio (SNR) and is more suitable for the hostile environment compared to DAS. When the current is injected into the DFB laser with a low frequency and a high frequency sinusoidal waveform, the intensity of the laser, *I_0_*(t), and the frequency of the laser, ν(t), can be modeled using Equations (2) and (3), [14] respectively.

(2) $$I_{0}\left(t\right)={\bar{I}}_{0}(1+i_{1,s}\sin\left(2\pi f_{s}t+\varphi_{1,s}\right)+i_{2,s}\sin\left(4\pi f_{s}t+\varphi_{2,s}\right)\;+i_{1,m}\sin\left(2\pi f_{m}t+\varphi_{1,m}\right)+i_{2,m}\sin\left(4\pi f_{m}t+\varphi_{2,m}\right))$$

(3) $$v\left(t\right)={\bar{v}}_{0}+a_{1,s}\sin\left(2\pi f_{s}t+\emptyset_{1,s}\right)+a_{2,s}\sin\left(4\pi f_{s}t+\emptyset_{2,s}\right)\;+a_{1,m}\sin\left(2\pi f_{m}t+\emptyset_{1,m}\right)+a_{2,m}\sin\left(4\pi f_{m}t+\emptyset_{2,m}\right)$$

Here, the subscripts *s* and *m* represent the characteristic changes of intensity or frequency due to the scan and modulation, respectively. ${\bar{I}}_{0}$ is the mean intensity; $i_{1}$ and $i_{2}$ are the first- and second-order mean-normalized intensity amplitude, respectively; f [Hz] is the user-specified frequency; $\varphi_{1}$ and $\varphi_{2}$ are the absolute phase shift of the first- and second-order intensity; similarly, $\emptyset_{1}$ and $\emptyset_{2}$ are the absolute phase shift of the first- and second-order frequency; ${\bar{v}}_{0}$ (cm^−1^) is the center optical frequency of the laser. $a_{1}$ and $a_{2}$ [cm^−1^] are the first- and second-order scan or modulation depth.

The low frequency current scans the majority of absorption lineshape, while the high frequency modulation current transfers the absorption information to the harmonics. In order to acquire absorption information from harmonics, the most widely used method is the 2nd harmonics normalized by 1st harmonics, namely WMS-*2f/1f*. In this study, the harmonics were obtained using the approach presented by Goldenstein et al. [14]. The transmitted light signal is multiplied by a reference cosine and sine wave at 2*f*_m_, respectively, which are then passed through the low-pass filter to obtain the corresponding *X_2f_*(*t*) and *Y_2f_*(*t*) components. The *X_1f_*(*t*) and *Y_1f_*(*t*) can be obtained in a similar way, where the frequency of the reference signal is changed to 1*f*_m_. The WMS-*2f/1f* signal intensity can be calculated by eq.4, which is a function of the integrated absorbance area, lineshape function and laser characteristics [14].

(4) $$WMS_{2f/1f}(t)=\sqrt{X_{{}_{2f}}^{2}(t)+Y_{{}_{2f}}^{2}(t)}/\sqrt{X_{{}_{1f}}^{2}(t)+Y_{{}_{1f}}^{2}(t)}$$

**Figure 1:**
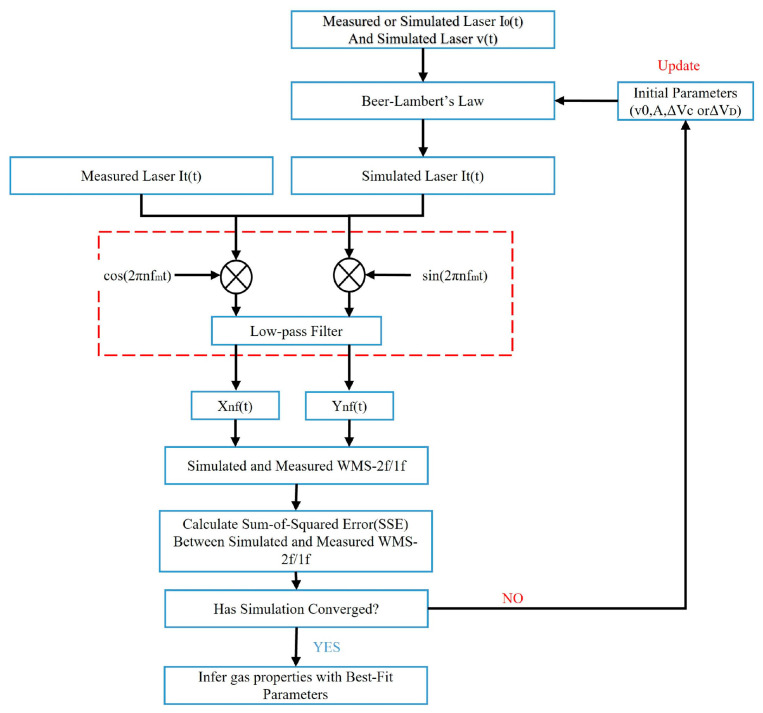
Flow chart of scanned-WMS-*2f/1f* spectral fitting routine. The guess values of initial parameters are firstly employed in the iteration. If the SSE is smaller than the predetermined value when the iteration converges, the outputs of the iteration are used to infer gas properties. Otherwise, the initial parameters have to be updated and repeat the iteration.

In this scheme, the transition center frequency ($v_{0}$), the integrated absorbance area (*A*) and the lineshape width (Δν) are employed as free parameters to fit the simulated WMS-*nf/1f* spectral profile to the measured one. The iteration fitting routine is shown in Figure 1. When the minimum sum-of-squared errors (SSE) between the simulation and measured WMS-*2f/1f* meet the user-specified requirement, the best fitting parameters are employed to determine the gas property. An intelligent guess for initial parameters would reduce the update times dramatically. However, it is difficult to obtain the intelligent guesses for the fitting routine when under an unknown circumstance. The less you know about the measurement situation, the harder you acquire appropriate guesses. One should note that the lineshape width should be dependent on both the collisional width and Doppler width. Nevertheless, in order to reduce the fitting complexity, only the collisional width is floated and the Doppler width is fixed at a given temperature. If the inferred gas temperature deviates far away from the given temperature, an iteration between Doppler width and fitting procedure may be needed.

### 2.2. Orthogonal Test Method

Orthogonal test method (OTM) is used to study several factors and levels. Based on the appropriate orthogonal table, it can conduct tests with optimum parameters to reduce test times and curtail the test period. The factors are referred to the parameters which influence the final result in a specified experiment, while the levels are equal to the maximum number of the values that any single factor can be set as [26].

In this study, the WMS-*2f/1f* spectral profile is affected by the initial values, namely the integrated absorbance area (*A*), center frequency ($v_{0}$) and collisional width (${\Delta \nu }_{c}$). If the initial values are chosen inappropriately, the final value of SSE may not satisfy the requirement of the predetermined SSE after a certain amount of iteration, even though the iteration converges. Sequentially the initial values have to be updated and the iteration process is repeated to meet the requirement. Normally, the farther the initial parameters deviate from the true gas properties, the more times they need updated, which is time-consuming and puts challenge to the hardware device. On the contrary, when the improved initial values are employed, it can decrease the initial-parameters-updating times and consequently curtail the period of the fitting routine.

Therefore, the *A*, $v_{0}$ and ${\Delta \nu }_{c}$ are set as three factors in an orthogonal experiment to determine the optimal initial values. The levels of each parameter are selectively some typical values of parameters according to specific conditions. In this study, the three levels of *A* are 0.001, 0.002 and 0.003. The three levels of $v_{0}$ are 7444.35, 7444.37 and 7444.39 cm^−1^. Those of ${\Delta \nu }_{c}$ are selected as 0.02, 0.05 and 0.08 cm^−1^. All the factors of levels are decided by roughly calculating, and are not required to be specific. The chosen factors and their corresponding levels are shown in Table 1.

## 3. Experimental Validation

**Figure 2:**
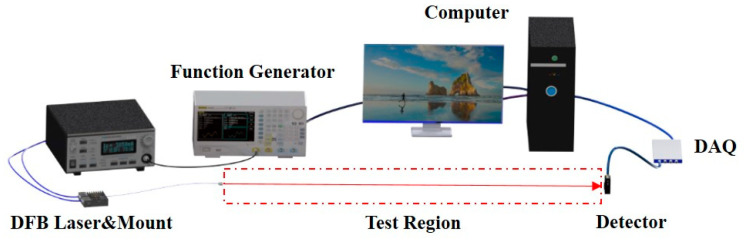
The schematic of the experimental setup.

The schematic of the experimental setup is shown in Figure 2. To validate the performance of the optimized initial parameters determined by OTM, a DFB tunable diode laser (NEL) near 1343 nm was used to probe the vapor temperature based on scanned-WMS-*2f/1f* at room temperature (291K, 1 atm). The H_2_O mole fraction was 0.7% measured by a hygrometer. A function generator (RIGOL 4102) controlled by the LabVIEW was used to produce a slow sinusoidal waveform (*f*_s_ = 100 Hz) and a fast one (*f*_m_ = 10 kHz). The combined waveform was then delivered to a laser controller (Arroyo 6305), which can change the output intensity and wavelength of a DFB laser via temperature and current. The light from the laser passed through the test region, whose optical length (*L*) was 137 cm and was directed upon the detector (Thorlabs, PDA20CS2). The transmitted signal was recorded by a USB DAQ (NI 9223) with a 1 Msa/s sampling rate. The raw signal was then processed in a computer to obtain the measured harmonics’ signals. It must have been mentioned that the frequency characteristics (v(t)) of this laser has been conducted prior to the experiment.

## 4. Results and Discussions

### 4.1. Optimized Initial Parameters Obtained by OTM

**Figure 3:**
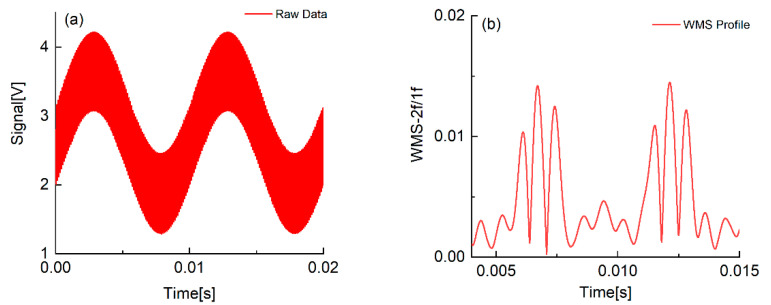
The raw detected signal (**a**) and the corresponding WMS-*2f/1f* spectral profile (**b**).

The partially raw signal and two typical sets of WMS-*2f/1f* spectral profiles are shown in Figure 3. Although the absorption of H_2_O is very weak in the room condition, the vapor properties can also be inferred from the WMS-*2f/1f* profile with the strategy introduced in Section 2.1.

To gain the optimized initial parameters and shorten the fitting routine period, a parameter-optimization procedure based on OTM using orthogonal table L_9_ (3^4^) was performed. Since the goodness of fitting between the simulated and measured WMS-*2f/1f* profile is indicated by the SSE, it is appropriate to employ the value of SSE to evaluate the fitting performance of different initial parameters after the given updating times. The different combinations of three factors with three levels (in Table 1) based on the orthogonal table were used as the initial parameters to fit the measured WMS-*2f/1f* profile, and the minimal SSE was calculated after the initial parameters updated 50 times, as shown in Table 2. In the fitting routine, the Levenberg-Marquardt algorithm was employed. It is obvious that the values of SSE vary dramatically with different initial parameters. The bigger the value is, the worse is the fitting performance. Moreover, it is necessary to update the initial parameters with more times to obtain satisfactory results. Take the test 8 for example; the value of SSE is as high as 0.9561091, which indicates the fitting effect is not satisfying. Therefore, the initial parameters of test 8 have to be updated with more times to improve the fitting performance.

It necessitates further analysis of the orthogonal test results to locate the optimal initial parameters via range analysis. Range analysis is a statistical method to determine the factors’ sensitivity to the experimental result according to the orthogonal experiment. Range is defined as the distance between the extreme values of the data. The greater the range is, the more sensitive is the factor [33]. The summation of SSE from *i-level* for a specific *x-factor*,$K_{i}^{x}$, the average of $K_{i}^{x}$, $k_{i}^{x}$, and the range, *R^x^*, is calculated by Equations (5)–(7) [28], respectively. The results are listed in Table 3.

(5) $$K_{i}^{x}=\sum SSE\;\mathrm{from the level i and factor x}$$

(6) kix=Kix3

(7) $$R^{x}=\max(k_{1}^{x},k_{2}^{x},k_{3}^{x})-\min(k_{1}^{x},k_{2}^{x},k_{3}^{x})$$

According to the value of R in Table 3, it can be concluded that the SSE, namely, the performance of fitting, is primarily affected by the initial value of the integrated absorbance area among the three factors. However, it is reminded that the effects of the other two factors cannot be ignored. From the data in Table 3, it can be found that $k_{1}^{A}<k_{2}^{A}<k_{3}^{A}$, $k_{1}^{v_{0}}<k_{3}^{v_{0}}<k_{2}^{v_{0}}$ and $k_{3}^{∆v_{C}}<k_{2}^{∆v_{C}}<k_{1}^{∆v_{C}}$. This result implies that the optimal initial parameters are the combination of the integrated absorbance area, *A*, with Level 1, center frequency,$\;v_{0}$, with Level 1 and collisional width,$\;{\Delta \nu }_{c}$, with Level 3, namely, 0.001, 7444.35 and 0.08 cm^−1^, respectively.

### 4.2. Fitting Performance of the Optimized Initial Parameters

**Figure 4:**
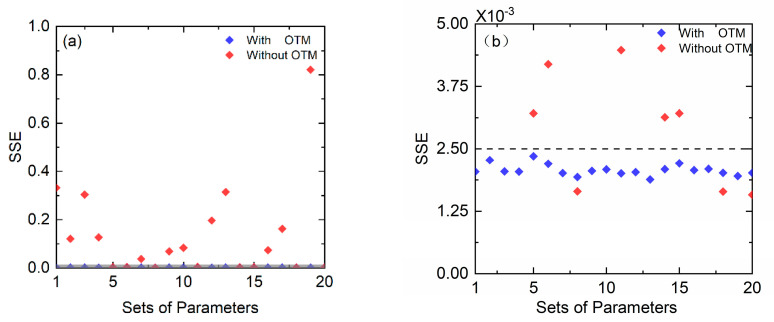
(**a**) The values of SSE between the simulated and measured WMS–*2f/1f* spectral profile. The red diamond indicates the minimal SSE of the fitting by the optimized initial parameters obtained from the orthogonal test method, while the red ones represent the minimal SSE of the fitting by random initial parameters. (**b**) presents the zoomed in view of the gray region in (**a**). It is evident that all the SSE from the optimized initial parameters are less than 0.0025, while it is only 3 sets for random initial parameters, as shown in (**b**).

With the optimized initial parameters obtained above, the fitting routine can meet the SSE requirement within less initial parameter-updating times and improve the fitting efficiency. To illustrate this benefit, 20 sets of random values around the optimized initial parameters and 20 sets of random initial parameters in the range from 0.001 to 0.003 for *A*, 7444.35 to 7444.37 cm^−1^ for $v_{0}$ and 0.02 to 0.08 cm^−1^ for ${\Delta \nu }_{c}$ were used to match the same measured WMS-*2f/1f* spectral profile. All the initial parameters were allowed to be updated no more than 50 times, and their respective minimal SSE were calculated. The final results are shown in Figure 4.

In this figure, the blue diamond indicates the minimal SSE of the fitting by the optimized initial parameters obtained with OTM, while the red ones represent the minimal SSE of the fitting by random initial parameters. In Figure 4a, it is apparent that all the minimal SSEs from the optimized parameters are buried in the gray layer. By contrast, the majority of the minimal SSEs from random initial parameters are outside the gray region. This characteristic implies that the optimized initial parameters obtained from the OTM can reach a smaller SSE in given updating times compared with the random ones. Furthermore, if the user-specified SSE is 0.0025, all the SSEs from optimized initial parameters can meet the requirement. However, only three sets of random initial parameters’ SSEs are smaller than the required SSE, as shown in Figure 4b. It means that those initial parameters, which do not satisfy the requirement, need more times to update their values.

**Figure 5:**
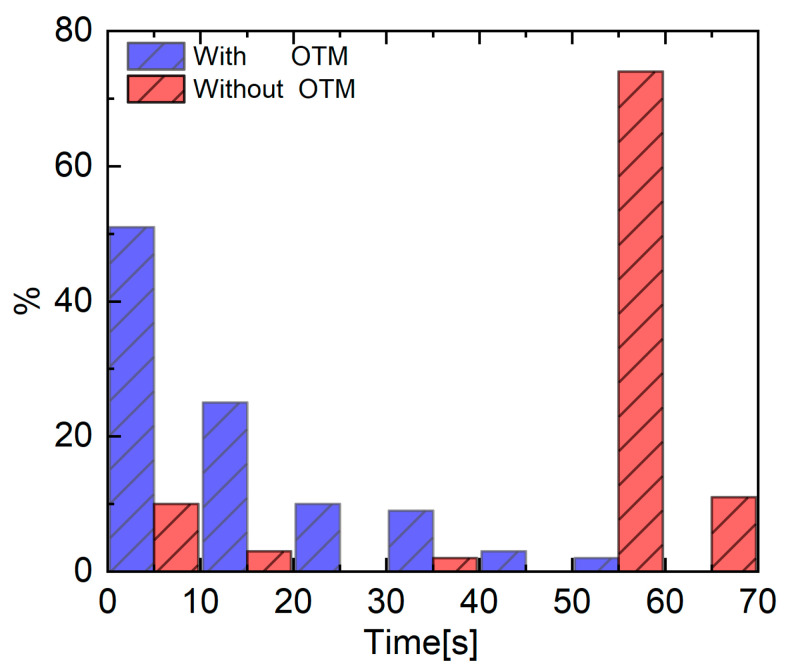
The time distribution comparison of the optimized initial parameters and the unimproved ones to fit the same measured WMS−*2f/1f* spectral profile.

Theoretically, as long as the initial parameters are updated frequently enough, they eventually can meet the SSE requirement to converge the fitting routine, no matter which values the initial parameters takes. However, it is rather time-consuming. To illustrate this characteristic visually, 100 sets of initial parameters were selected randomly around the optimized values obtained by OTM and from the same range as above, respectively, all of which were applied to match the same measured WMS-*2f/1f* spectral profile. As the Figure 5 shows, the blue bar represents the distribution of time the near optimized initial parameters took, and the red is the distribution of time cost by the unimproved initial parameters. It is obvious that the majority of the optimized parameters took less than 20 s to converge the fitting routine, accounting for near 80%. More specifically, many of them took even less than 5 s and no one was found beyond 60 s. Nevertheless, when fitting with the parameters without optimization, nearly 90% of the sets cost more than 50 s to obtain the same results. In addition, the sets of parameters costing below 20 s accounted for less than one fifth. The averagely consumed time with the optimized initial parameters was 10.3 s, while it was 49.5 s for the parameters without optimization, near 5 times longer than the former. Therefore, the optimized initial parameters can effectively curtail the period of fitting routine and accelerate gas property inference from the WMS-*2f/1f* spectral profile, which is helpful, especially when faced with a large amount of fitting data.

### 4.3. Temperature from the WMS-2f/1f

**Figure 6:**
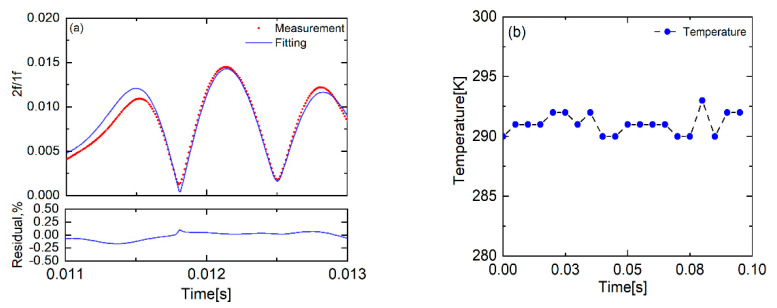
(**a**) The measured and the best-fit WMS−*2f/1f* spectral profile. (**b**) The inferred temperature in room conditions by the WMS−*2f/1f* strategy.

Figure 6a presents a single-scan measured WMS-*2f/1f* spectral profile of the H_2_O transition near 7444.35 cm^−1^ and the corresponding best-fit with the optimized initial parameters. In all fitting routines, ${\Delta \nu }_{D}$ was fixed at the value given by the known temperature and *A*, $v_{0}$ and ${\Delta \nu }_{c}$ were free parameters obtained by OTM. The simulated WMS-*2f/1f* spectral agrees well with the whole measurement. However, there exists a visible difference in the region of the left lobe, which may be caused by the low absorbance as stated in Section 4.1. However, this deviation does not influence the final gas property inference too much.

The integrated absorbance area from the scanned-WMS-*2f/1f* spectral fitting routine was used to deduce the vapor temperature with the known H_2_O mole fraction, 0.7%, measured by a hygrometer. Figure 6b shows the inferred temperature during 10 scan-cycles of the laser, namely 0.1 s. The WMS-*2f/1f* measured temperature was in good agreement with the value measured by K type thermocouple (291K). The slight variation of the WMS-*2f/1f* measured temperature was reasonable. The measurement uncertainty originates primarily from the fitting residual (<0.25% from Figure 6a),the hygrometer (~1%) and the reference linestrength (~2%) [34]. Therefore, the measured temperature uncertainty was about 2.25%. Note that the laser was scanned by a sinusoidal waveform with 100 Hz, so there existed absorption when scanned up or down in one cycle. Therefore, the measurement temporal resolution was increased to 200 Hz.

## 5. Conclusions

A strategy based on the orthogonal test method is proposed to optimize the initial parameters of the scanned-WMS-*2f/1f* spectral fitting routine. With the optimized initial parameters, the sum-of-squared errors (SSE) between the simulated and measured WMS-*2f/1f* profile can be greatly reduced in the given initial-parameters-updated times. Consequently, the fitting routine converges more efficiently. As stated before, on average, it took about 10 s to make the fitting routine converge with the optimized initial parameters. Meanwhile it needed 50 s to obtain the same results using the conventional method. When faced with a large amount of fitting data, it can save roughly 80% calculating time using this novel method. The measurement of gas temperature was performed by WMS in the controlled condition (T = 291K, X_abs_ = 0.7%, P = 1 atm). Moreover the inferred temperature with the optimized initial parameters was consistent with the controlled value.

## Figures and Tables

**Table 1:** The factors and levels in the Orthogonal Test.

Factors	Integrated Absorbance Area (*A*)	$\mathrm{Center}\;\mathrm{Frequency}\;(v_{0})$ [cm^−1^]	$\mathrm{Collisional}\;\mathrm{Width}\;({\Delta \nu }_{c})$ [cm^−1^]
Level 1	0.001	7444.35	0.02
Level 2	0.002	7444.37	0.05
Level 3	0.003	7444.39	0.08

**Table 2:** Orthogonal Array L_9_(3^4^) and the SSE.

Test Numbers		Factors		SSE
	Integrated Absorbance Area (*A*)	$\mathrm{Center}\;\mathrm{Frequency}\;(v_{0})$ (cm^−1^)	$\mathrm{Collisional}\;\mathrm{Width}\;({\Delta \nu }_{c})$ (cm^−1^)	
1	0.001	7444.35	0.02	0.0106581
2	0.001	7444.37	0.05	0.0015216
3	0.001	7444.39	0.08	0.0042488
4	0.002	7444.35	0.05	0.0034304
5	0.002	7444.37	0.08	0.0043295
6	0.002	7444.39	0.02	0.0602594
7	0.003	7444.35	0.08	0.7740496
8	0.003	7444.37	0.02	0.9561091
9	0.003	7444.39	0.05	0.8002515

**Table 3:** Analysis of the orthogonal results.

	Factors		
	Integrated Absorbance Area (*A*)	$\mathrm{Center}\;\mathrm{Frequency}\;(v_{0})$ (cm^−1^)	$\mathrm{Collisional}\;\mathrm{Width}\;({\Delta \nu }_{c})$ (cm^−1^)
$K_{1}^{x}$	0.016429	0.788138	1.027027
$K_{2}^{x}$	0.068019	0.961960	0.805204
$K_{3}^{x}$	2.530410	0.864760	0.782628
$k_{1}^{x}$	0.005476	0.262713	0.342342
$k_{2}^{x}$	0.022673	0.320653	0.268401
$k_{3}^{x}$	0.843470	0.288253	0.260876
$R^{x}$	0.837994	0.057940	0.081466

## Data Availability

The data presented in this study are available on request from the corresponding author.
